# Integrative metabolic and microbial profiling on patients with Spleen-yang-deficiency syndrome

**DOI:** 10.1038/s41598-018-24130-7

**Published:** 2018-04-26

**Authors:** Zhang Lin, Wu Ye, Xianpeng Zu, Haisheng Xie, Houkai Li, Yiping Li, Weidong Zhang

**Affiliations:** 1 School of Pharmacy, Shanghai Jiaotong University, Shanghai, 200000 China; 2 Department of Gastroenterology, Shanghai Municipal Hospital for Traditional Chinese Medicine, Shanghai, 200000 China; 3 School of Pharmacy, Second Military Medical University, Shanghai, 200000 China; 4 Centre for Chinese Medical Therapy and Systems Biology, Institute for Interdisciplinary Medicine Sciences, Shanghai University of Traditional Chinese Medicine, Shanghai, 201203 China

## Abstract

Gut microbiota is recognized as an indispensable “metabolic organ” that plays crucial roles in maintaining human health or initiating diseases. Spleen-yang-deficiency syndrome (SYDS) is a common syndrome of Traditional Chinese Medicine (TCM) clinic. It is a complex phenotype reflecting the overall changes of metabolism which are mainly caused by digestive disorders. However, little is known about the changes of gut microbiota and metabolism in patients with SYDS, as well as the crosstalk between gut microbiota and host metabolism. In the current study, an integrative metabolic and microbial profiling was performed on plasma, urine and feces from recruited SYDS and healthy individuals by using a LC-QTOFMS-based metabolomic and 16 s rRNA sequencing approaches. Our results showed a potentially significant contribution of gut dysbiosis to the metabolic disorders in SYDS. By integrating the differential gut bacteria with the metabolites, the results revealed some active bacterium of norank_f_*CFT112H7*, f_*lachnospiraceae* and *bacteroides* were closely involved in host mucosal integrity, bile acid metabolism and polysaccharides decomposition. Therefore, our results indicated the probable involvement of gut microbiota in mediating the metabolic changes, which warrants a further investigation on the role of gut microbiota in modulating the pathogenesis of SYDS.

## Introduction

In traditional Chinese medicine (TCM), “Spleen” is completely different with that of the spleen organ in western medicine. “Spleen” in TCM is associated with stomach that is responsible for food and fluid assimilation and transformation into usable nutrients for the body^1^. Thus, “Spleen dysfunction” in TCM is a panel of comprehensive syndromes that are involved with various disorders in digestive and circulatory systems^2–4^. The typical characters of “Spleen dysfunction” include poor appetite and digestion, fatigue, and bleeding disorders. Spleen-deficiency syndrome (SDS) is a typical syndrome of TCM, which is characterized by poor appetite, fullness and sleepiness after eating, fatigue, pale face and tongue, weight loss, and loose stools^5^. Spleen-yang-deficiency syndrome (SYDS) is one of the most common types of SDS. Patients with SYDS are usually diagnosed with abnormalities in the gastroenterological system such as diarrhea, chronic gastroenteritis or hepatitis and *candida* infection in western medicine^6–9^. However, to date the biological mechanisms underlying SYDS are largely unknown.

In recent years, humans have been recognized as ‘superorganisms’ due to the fact that the human body is composed of both human and microbial cells. The commensal bacteria in the gut play critical roles in maintaining human health and mediating disease development because, as a superorganism, we carry two sets of genes namely the host genome and those encoded by microbiota^10–13^. The gut microbiota is thus very important to digestive system functions of humans because of the enrichment of genes involved in polysaccharide utilization derived from gut microbiota coded genomes^14–16^. As described in TCM theory, SYDS is a complex phenotype, which reflects the overall changes in metabolic processed or networks mainly caused by digestive system disorders^3,5,17^. Thus, theoretically, gut microbiota dysbiosis may well contribute to the development of SYDS^18^, but little evidence for this hypothesis has been acquired in the clinic.

The gut microbiota not only help the host to digest some complex compounds such as cellulose and polysaccharides, it also secretes active compounds (e.g. short chain fatty acids, phenyl/indole related products, bile acids and so on) into biofluids such as blood, urine and feces to take part in the systematic circulation and metabolism. These microbial metabolites can have diverse beneficial or toxic biofunctions that are vital to the host health and the development of diseases. In this regard, the gut microbiota has also been regarded as a “metabolic organ” in the human body. The importance of gut flora to host metabolism can be explained by examples that animals with identical genes may have diverse metabolic phenotypes when they have different gut flora in their intestinal tract^19^. Metabolomics is the scientific study of chemical processes, which measures all the changes of endogenous small molecular metabolites including those generated by gut microbiota metabolism^20–23^. This is a scientific method consistent with the systematic concept of TCM. Therefore, in order to characterize the global characteristics of TCM syndrome, it is indispensable to combine research into gut microbiota with metabolic profiling.

To characterize the metabolic and gut microbiota-related changes which are associated with the pathophysiology of patients with SYDS, in the current study, an integrative LC-QTOF-MS based-metabolic and 16 s rDNA based microbial profiling was performed among patients with SYDS, as well as matched healthy volunteers. Our current results revealed significant differences between patients with SYDS and healthy volunteers in both metabolic (plasma and urine) and microbial profiles. Moreover, the correlation analysis between differential metabolites and bacteria revealed significant crosstalk between the gut microbiota and host metabolism in SYDS patients. These results suggested the gut microbial involvement in TCM syndromes such as SYDS, and the modulation of gut microbiota may be a promising strategy for therapy on patients with TCM syndromes.

## Materials and Methods

### Recruitment of patients with SYDS and healthy volunteers

All of the volunteers were recruited from May to December in 2016 in Shanghai Municipal Hospital for Traditional Chinese Medicine, Shanghai, China. Their baseline clinical information is presented in Table 1. The SYDS group were diagnosed based on the ‘Guidelines for the Clinical Research of Chinese Medicine New Drugs’^24^. The dominant symptoms are: (a) a pale tongue with a thin-white coating, (b) poor appetite, (c) abdominal distension, and (d) loose stools or diarrhea. The secondary symptoms are: (a) thinness, (b) weakness, and (c) a weak pulse. Possession of a pale tongue with a thin-white coating is essential for a diagnosis of Spleen-deficiency-syndrome pattern (SDP), and this should be combined with two of the other three dominant symptoms, or combined with one of the others and at least two of the secondary symptoms. Syndrome pattern differentiation was performed by two of the authors (Wu Ye and Zhang Lin), and disagreement was resolved by discussion with Yiping Li. Healthy volunteers were interviewed by one of the authors (Wu Ye) who is a certificated physician in TCM. The volunteers were free of any diseases during study including infectious and inflammatory diseases, psychiatric and serious somatic diseases, and dyslipidemia, pregnant or breastfeeding. They also had no apparent TCM syndrome patterns with normal tongue and pulse. In total, 30 patients with SYDS (20 female/10 male) and 30 healthy volunteers (16 female/14 male) were included. The study followed the guidelines of the Declaration of Helsinki and Tokyo for Humans, and was approved by the Institutional Ethics Committee at Shanghai Municipal Hospital for Traditional Chinese Medicine. All subjects were informed consent and agreed to use the resulting information in peer reviewed medical publications and study participation.

**Table 1:** Clinical information for SYDS patients and healthy volunteers.

Group	SYDS	Health	P-value
Number	30	30	
Age/year	36.1 (22.0–69.0)	43.0 (28.0–66.0)	0.559
Gender (Female/Male)	20/10	16/14	0.279
Clinical diagnosis	Chronic gastritis, 6 Others 24	—	

### Gut microbiota profiling

#### Stool sampling

In the morning following overnight fasting, a 5 g fresh stool sample was collected from each individual into a new 2 mL sterile centrifuge tube. Then the samples were snap-frozen in liquid nitrogen and stored at −80 °C for subsequent analysis.

#### DNA extraction

Microbial DNA was extracted from fecal samples using the E.Z.N.A.® soil DNA Kit (Omega Bio-tek, Norcross, GA, U.S.) according to the manufacturer’s guidelines. The final DNA concentration and purification were determined using a NanoDrop 2000 UV-vis Spectrophotometer (Thermo Scientific, Wilmington, USA). The quality of the DNA was checked using 1% agarose gel electrophoresis.

#### PCR amplification of 16S rRNA V3–V4 regions

The V3–V4 region (338F-806R) of 16S rRNA was amplified by PCR with the primers 338 F (5′-ACTCCTACGGGAGGCAGCA-3′) and 806 R (5′-GGACTACHVGGGTWTCTAAT-3′). The PCR components were as follows: 5 × FastPfu Buffer (4 μL), 2.5 mMdNTPs (2 μL), forward primer (5 μM, 0.8 μL), reverse primer (5 μM, 0.8 μL), FastPfu polymerase (0.4 μL), and template RNA (10 ng). The reaction volume was brought up to 20 μL with ddH_2_O. The PCR conditions were 2 min at 95 °C followed by 27 cycles of 30 sec at 95 °C, 30 sec at 55 °C, and 45 sec at 72 °C, and finally 10 min at 72 °C. The resulting PCR products were extracted from a 2% agarose gel and further purified using an AxyPrep DNA Gel Extraction Kit (Axygen Biosciences, Union City, CA, USA) and quantified using QuantiFluor™-ST (Promega, USA) according to the manufacturer’s protocol.

#### High-throughput sequencing

Purified amplicons were pooled in equimolar concentrations and paired-end sequenced (2 × 300) on an Illumina MiSeq platform (Illumina, San Diego, USA) according to the standard protocols in Majorbio Bio-Pharm Technology Co. Ltd. (Shanghai, China).

#### Bioinformatics analysis

Raw fastq files were quality-filtered by Trimmomatic and merged by FLASH with the following criteria: (i) the reads were truncated at any site receiving an average quality score <20 over a 50 bp sliding window. (ii) sequences whose overlap was longer than 10 bp were merged according to their overlap with mismatch (no more than 2 bp). (iii) sequences of each sample were separated according to barcodes (exactly matching) and primers (allowing for 2 nucleotide mismatches); reads containing ambiguous bases were discarded.

Operational taxonomic units (OTUs) were clustered with a 97% similarity cut-off using UPARSE (version 7.1 http://drive5.com/uparse/) with a novel ‘greedy’ algorithm that performs chimera filtering and OTU clustering simultaneously. The taxonomy of each 16 S rRNA gene sequence was analyzed by the RDP Classifier algorithm (http://rdp.cme.msu.edu/) against the Silva (SSU123) 16 S rRNA database using a confidence threshold of 70%.

The calculations of the rarefaction curve, richness index, diversity index and Bray-Curtis distance *et al*. were performed with the mother^25^ built-in commands. Lefse software^26^, which performs a nonparametric Wilcoxon sum-rank test followed by linear discriminant analysis (LDA) coupled with measurements to assess the effect size of each differentially abundant taxon, was used to search for taxons in which the relative abundance was significantly different among the various populations. Alpha = 0.05 was used in the Wilcoxon rank sum test and the log value for the LDA analysis was set to be < 2.0. All the data analysis for gut microbiota was finished in online “i-sanger” (http://www.i-sanger.com/) developed by Majorbio Bio-Pharm Technology Co. Ltd.

### Metabolic profiling

#### Plasma/urine collection and preparation

The biofluids (blood, urine) samples were collected at the same time point together with stool samples from each recruited volunteer. For plasma metabolomics, 2 mL of venous blood was drawn from each individual into a K_2_EDTA anticoagulation tube. Each tube was gently inverted five times to ensure proper mixing of blood with the anticoagulant. The blood was then centrifuged for 10 min (3,000 g, 4 °C) to obtain plasma and subsequently divided into 100 μL aliquots and stored at −80 °C. Before LC analysis, the plasma samples were thawed at 4 °C for 30 min. To each aliquot, 300 μL of methanol was added and mixed for 30 s on a vortex. The sample was centrifuged for 15 min (14,000 rpm, 4 °C). Finally, the supernatant was transferred to auto-sampler vials and stored at −80 °C for LC-MS analysis.

For urine metabolomics, 3 mL of urine was collected into a tube from each individual and stored at −80 °C. Before LC analysis, it was thawed and pre-processed as for the plasma samples (vide supra).

#### Chromatography

For plasma profiling, chromatographic separation was performed on a 1.7 μm, 2.1 mm × 100 mm ACQUITY UPLC®BEH C_18_ column (Waters, USA) using an ACQUITY Ultra Performance LC system (Waters corp., Milford, MA, USA) equipped with a binary solvent delivery system, an auto-sampler, and high temperature column oven. The column was maintained at 40 °C. The flow rate was set at 0.4 mL/min. The sample injection volume was 2.5 µL. Solvent A was water mixed with 0.1% formic acid, and solvent B was acetonitrile mixed with 0.1% formic acid. The gradient elution programmes were: 0 min 5% B, 0–6 min 100% B, 6–8 min 100% B, 8–9 min 90% B, 9–14 min 80% B, 14–14.1 min 5% B.

For urine profiling, chromatographic separation was performed on a 1.8 μm, 2.1 mm × 100 mm HSS T3 column (Waters, USA) using an ACQUITY Ultra Performance LC system (Waters corp., Milford, MA, USA) equipped with a binary solvent delivery system, an auto-sampler, and high temperature column oven. The gradient elution programmes were: 0 min 5% B, 0–9 min 100% B, 9–12 min 100% B, 12–12.1 min 5% B. Other parameters for LC separation were the same as those used in plasma metabolomics.

#### Mass spectrometry

MS detection was acquired on a SYNAPT G2-Si high-resolution mass spectrometer (Waters Corp., Milford, MA, USA) equipped with an electrospray ion (ESI) source. The desolvation gas was set at 900 L/h at a temperature of 500 °C. The cone gas was set at 50 L/h. The source temperature was set at 120 °C. The capillary and cone voltage were optimized at 2 kV and 49 V, respectively. Full scan spectra were performed between 50–1200 m/z with a 0.2 s scan time and a 0.1 s interscan delay. Positive and negative ion modes were both acquired in mass detection. All analyses were acquired using the lock spray to calibrate the system. Leucine encephalin was used as the lock spray at a concentration of 1 ng/μL and a flow rate of 3 μL/min, generating a reference ion for the positive ion mode ([M + H]^+^  = 556.2771) and negative ion mode ([M-H]^−^ = 554.2615). This technique enables the generation of mass information with high accuracy and precision for the identification of known or unknown compounds. Additionally, mass spectrometry elevated energy (MS^E^) collection was applied for additional compound identification. This technique provides parallel alternating scans for acquisition at either low collision energy to obtain precursor ion information or the ramping of high collision energy to obtain full-scan accurate mass fragments, precursor ions and neutral loss information.

#### Data pre-processing, multivariate statistical analysis and metabolite identification

The raw plasma/urine LC-MS data were both pre-processed using Waters Progenesis QI 2.0 software (Nonlinear Dynamics, Newcastle, U.K.). Progenesis QI can provide an accurate measurement of the compounds in the bio-samples and facilitate flexible comparisons across runs. It included steps of importing data, reviewing alignment, experiment design setup, picking peaks, identifying and reviewing compounds and performing compound statistical analysis. Then, the data were exported into EZinfo 3.0 for multivariable statistical analysis. The multivariate statistical analysis (MVA) of principal component analysis (PCA) and orthogonal partial least square-discriminant (OPLS-DA) models were both applied to observe the classifications for different groups in score plots. Next, to find significant metabolites contributing to the classifications, ions with VIP > 1.5 (VIP = variable importance in the projection) were picked out from the OPL-DA loading plots. Then univariate analysis of *t*-test and fold-change were also applied to these ions. Ions with *P*-values < 0.05 and fold-changes > 1.5 (or < 0.67)) were finally regarded as differentiated metabolite ions. Then they were structurally identified and interpreted based on searches of their accurate masses in metabolomic associated databases: METLIN (www.metlin.scripps.edu), HMDB (www.hmdb.ca) and KEGG (www.genome.jp/kegg). Lastly, the isotopic distribution, retention time and fragments of commercial standards were further confirmed for the metabolites of interest.

#### Pathway analysis using MetaboAnalyst software

MetaboAnalyst 3.0 (http://www.metaboanalyst.ca/) is a comprehensive tool suite for metabolomics data analysis. This platform currently supports a total of 1,600 pathways and visualization for 21 model organisms, including human, mouse, rat, cow, chicken, zebrafish, malaria, budding yeast, and so on^27^. The module of ‘Metabolic pathway analysis’ was applied in the present study to explore the associated metabolic pathways for all the differentiated metabolites.

#### Correlation analysis for microbial taxonomy and metabolite biomarkers

Covariation between UPLC-MS-based metabolite biomarkers (plasma and urine) and 16 s rRNA sequence data are presented in the form of a heatmap diagram based on Spearman’s correlation coefficient. At the genus level, the correlation coefficient of R value was color-mapped onto the gut microbiota, showing how correlated each gut microbiota was with the identified metabolites. R values are presented in different colors, and the color card was the color partition of different r values (0.01 < p ≤ 0.05 *, 0.001 < p ≤ 0.01 **, p ≤ 0.001 ***). Next, the metabolic associations of each of the well-correlated members of the gut microbiome (e.g., r > 0.3 and < −0.3) were visualized in the form of cross-correlation maps, which displayed both positive (in red line) and negative (in green line) connections.

## Results

### Microbial profiling of SYDS patients and healthy volunteers

#### Sample size and sequencing depth

A total of 2,263,595 high quality reads were acquired from the 60 stool samples with averaged 37,727 sequences for each sample. After quality and chimera checking, 860 OTUs were determined (with 97% similarity). The Shannon-rarefaction curve (Fig. S1 in the supplementary material) revealed the suitable and sufficient sequencing depth of our study.

#### Compositional changes of gut microbiota in SYDS patients

**Figure 1:**
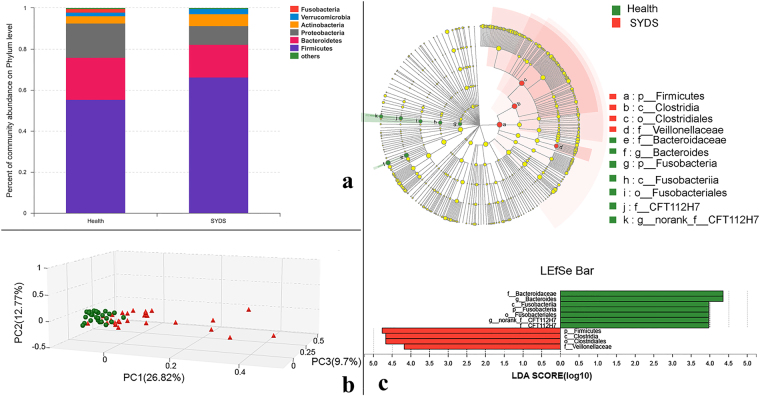
(**a**) microorganism community structures at the phylum level for healthy individuals and SYDS patients, different colors represent different bacteria at phylun level; (**b**) principal coordinates analysis (PCoA) of the microbial community for SYDS (in red) and healthy groups (in green); **(c**) different gut microbiota analysis for SYDS and healthy groups using Lefse software (linear discriminant analysis (also called LDA) coupled with effect size measurements). The abundances of taxa at the phylum, class, order, family, and genus levels were compared between the two groups. Taxa enriched in the SYDS group of patients are indicated by a positive LDA score (red), and taxa enriched in healthy individuals have a negative score (green). Only taxa meeting an LDA significant threshold of 4.0 are shown. For taxa, which were defined as unclassified, no rank or uncultured, the name of a higher taxon level was given before its taxon abbreviation.

Bacterial community structures in different groups are presented at the phylum level in Fig. 1a. The abundance is shown in terms of the percentage of the total number of sequences for each sample. *Firmicutes* had the highest abundance in most samples (with an average relative abundance of 60.97%). Additionally, genera that had relatively high abundances (>1.0%) were followed by *Bacteroides* (18.13%), *Proteobacteria* (13.01%), *Actinobacteria* (4.50%) and *Verrucomicrobia* (2.25%). As observed, the abundance of *Firmicutes* and *Bacteroides* were the two major categories, and the abundance of *Firmicutes* was largely increased while *Bacteroidetes* were decreased in SYDS patients compared to the healthy group. In recent reports, a higher *firmicutes*-to-*bacteroidetes* ratio was identified with energy metabolism dysfunction in both obese rats and humans^28–30^.

#### Beta diversity analysis

Further, to analyze whether the structure of the bacterial community in the gut microbiota differs between SYDS patients and healthy individuals, principal coordinates analysis (PCoA) in 3D view based on the Bray-Curtis distance was performed as shown in Fig. 1b. The results revealed that there were significant differences in the gut microbial community structure for the two groups regarding the first three principal component scores, which accounted for 49.29% (PC1 = 26.82%, PC2 = 12.77% and PC3 = 9.7%) of the total variations. Moreover, larger differences among SYDS samples (shown in red) were observed across the space indicating larger inter-individual variations among SYDS patients caused by a different pathological status.

#### Microbial differences between SYDS patients and healthy volunteers

To explore the differences in gut microbiota between SYDS patients and healthy controls, a Lefse difference analysis of taxon abundance was applied in our study. As shown (Fig. 1c), the top differences (LDA score > 4.0) in gut microbiota (from phylum to genus level) between SYDS patients and healthy controls were identified. In the SYDS patients, the relative abundances of a: p_*firmicutes*, b: c_*clostridia*, c: o_*clostridiales*, d: f_*veillonellaceae* were higher than those in the control group. In the healthy population, the abundance of e: *f_bacteroidaceae*, f: g_*bacteroides*, g: p_*fusobacteria*, h: c_*fusobacteriia*, i: o_*fusobacteriales* in the gut microbiota was higher compared to those in SYDS patients. As discussed above, the increase in the number of *firmicutes* bacteria in the gut mainly contributed to energy dysfunction in the SYDS group. Since 95% of the bacteria in *firmicutes* are *clostridia*, studies have shown that specific bacteria of *clostridium* cluster IV, *clostridium* cluster XIVa and cluster XVI in *clostridia* mainly contributed to energy metabolism disorders in *firmicutes*^31–33^.

### Metabolic profiling of SYDS patients and healthy volunteers

#### Pattern recognition analysis and differential metabolites identification

Metabolomics is a global approach to measure changes in endogenous compounds in biofluids or tissues, which are the end products of cellular processes. It is consistent with the overall concept and theory of TCM syndrome, which was applied to profile the plasma and urine samples to decipher the global changes of metabolic phenotype (metabotype) for the recruited populations.

**Figure 2:**
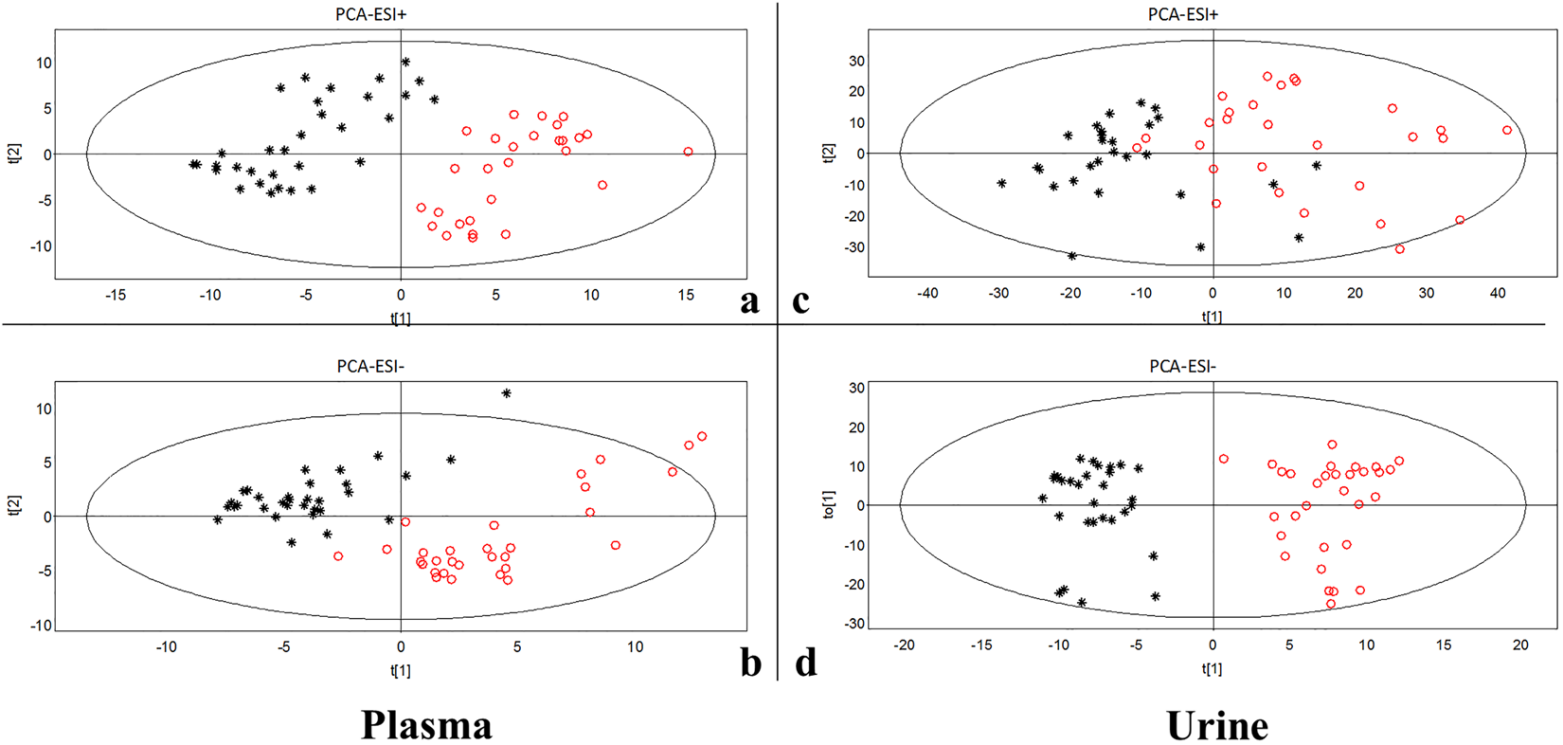
PCA score plots showing the distributions of plasma (**a**,**b**) and urine (**c**,) samples detected in both ESI + and ESI- modes for the SYDS patients (in red circle) and healthy individuals (in black star).

For plasma samples, SYDS patients were clearly separated from those in the healthy group (in black) in PCA (Fig. 2) and OPLS-DA score plots either in ESI + or ESI- modes (Fig. S2), indicating that the SYDS patients have totally different metabolic plasma characteristics from healthy individuals. In urine samples, the obvious separated distributions were also easily observed for the two groups in PCA (Fig. 2) and OPLS-DA score plots (Fig. S2). These results implied that the metabotypes in SYDS group were rather different from those in the healthy group.

Next, in order to identify the significant ions contributing to the classifications between the studied groups, multivariate and univariate statistical analysis were both applied for variables selection from OPLS-DA loading plots. Finally, some variables in plasma (Table 2) and urine (Table 3) were successfully identified as differentiated metabolites in the SYDS group.

**Table 2:** Differential metabolites in plasma between SYDS patients and healthy volunteers.

RT/min	Exact mass	Metabolites	Formula	Fold change SYDS/Health	Related pathway
7.79	284.295	Sphinganine	C18H39NO2	−4.9**	Sphingolipid metabolism
2.46	766.6589	Galactosylceramide(d18:1/22:0)	C46H89NO8	9.4**	Sphingolipid metabolism
5.46	379.2484	Sphingosine 1-phosphate	C18H38NO5P	−2.0**	Sphingolipid metabolism
5.64	380.2574	Dihydrosphingosine 1-phosphate	C18H40NO5P	−2.0**	Sphingolipid metabolism
1.17	133.0141	L-malic acid	C4H6O5	−2.4**	TCA cycle
1.74	117.0191	Succinic acid	C4H6O4	−3.8**	TCA cycle
1.75	261.0582	Citric acid	C5H6O4	5.9**	TCA cycle
1.12	349.0888	N-acetyl-L-aspartic acid	C6H9NO5	−1999.7**	Alanine, aspartate and glutamate metabolism
1.35	307.0346	dUMP	C9H13N2O8P	−2.7**	Pyrimidine metabolism
3.18	240.9765	3-mercaptopyruvic acid	C3H4O3S	−619.1**	Cysteine and methionine metabolism
1.43	263.1242	4-hydroxyproline	C5H9NO3	−∞**	Arginine and proline metabolism
1.12	387.1513	2-methylhippuric acid	C10H11NO3	+∞**	Fatty acid beta-oxidation
2.16	217.0287	Hypotaurine	C2H7NO2S	55.3**	Taurine and hypotaurine Metabolism
5.14	585.2700	Bilirubin	C33H36N4O6	−3.1**	Porphyrin and chlorophyll metabolism
1.06	181.0712	Sorbitol	C6H14O6	−2553.2**	Fructose and mannose metabolism
1.09	321.0946	4,6-dihydroxyquinoline	C9H7NO2	−5.6**	Tryptophan metabolism
1.35	89.0244	L-lactic acid	C3H6O3	1.8**	Glycolysis/Gluconeogenesis
5.46	317.2465	Tetrahydrodeoxycorticosterone	C21H34O3	−7.5*	Steroid hormone biosynthesis

*p < 0.05, **p < 0.01.

**Table 3:** Differential metabolites in urine between SYDS patients and healthy volunteers.

RT/min	Exac mass	Metabolites		Formula	Fold change SYDS/Health	Related pathway
5.42	279.2322	Gamma-linolenic acid		C18H30O2	23.6**	Linoleic acid metabolism
12.79	884.6116	PC (44:9)		C52H86NO8P	3.8**	Linoleic acid metabolism
4.05	333.2053	Prostaglandin E2		C20H30O4	−2.9*	Arachidonic acid metabolism
3.63	190.0502	5-Hydroxyindoleacetic acid		C10H9NO3	7.8**	Tryptophan metabolism
1.66	197.0667	Serotonin		C10H12N2O	2.0*	Tryptophan metabolism
1.90	211.0820	N-methylserotonin		C11H14N2O	−2.6*	Tryptophan metabolism
3.16	166.0569	4-Guanidinobutanoic acid		C5H11N3O2	5.6*	Arginine and proline metabolism
2.26	141.0003	3-Methylthiopropionic acid		C4H8O2S	13.1**	Cysteine and methionine metabolism
1.65	100.0397	L-threonine		C4H9NO3	−2.3*	Glycine, serine and threonine metabolism
2.66	425.1399	Chitobiose		C15H26N2O12	4.3*	Amino sugar and nucleotide sugar metabolism
4.21	103.0025	Hydroxypyruvic acid		C3H4O4	9.1*	Glycine, serine and threonine metabolism
2.12	179.0558	D-mannose		C6H12O6	−3.0**	Galactose metabolism
4.00	313.0388	dIMP		C10H13N4O7P	18.4*	Purine metabolism
1.62	413.0257	dIDP		C10H14N4O10P2	−2.5*	Purine metabolism

*p < 0.05, **p < 0.01.

#### Key metabolic pathway analysis for plasma/urine differential metabolites

**Figure-3:**
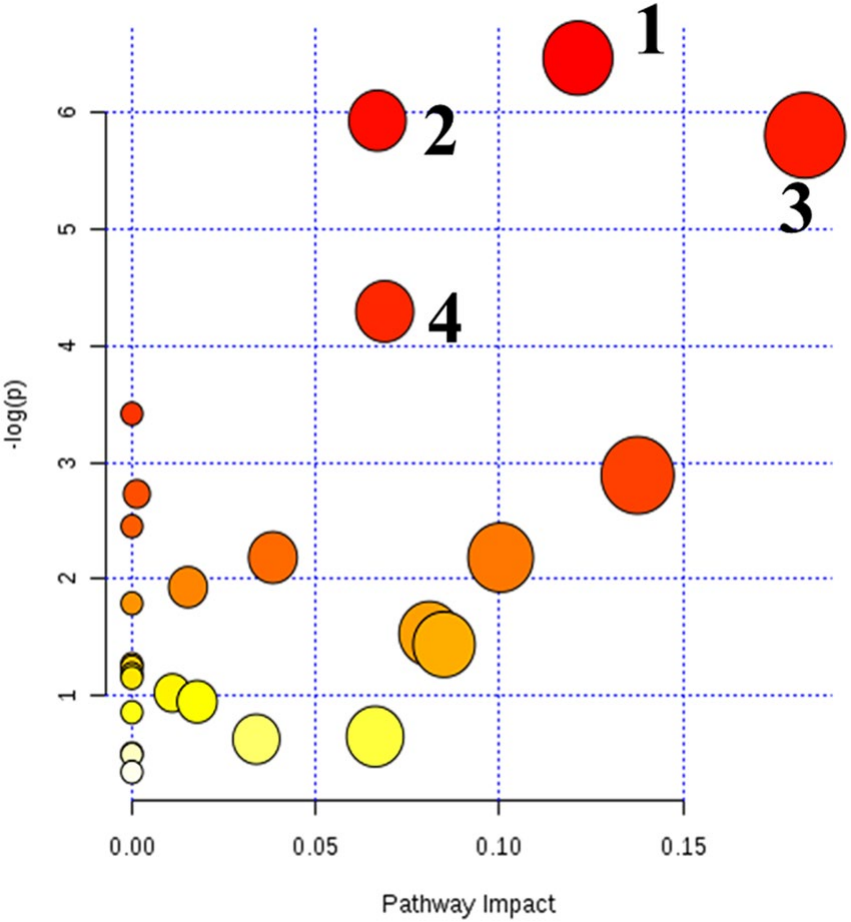
Summary of plasma and urine pathway analysis using Metaboanalyst. All the matched pathways are displayed as circles. The color and size of each circle was based on the p-value and pathway impact value, respectively. The smaller p value means the pathway with higher levels of significance. The most significant pathways include: 1, citrate cycle; 2, glyoxylate and dicarboxylate metabolism; 3, sphingolipid metabolism (p < 0.01); 4, tryptophan metabolism; 5, alanine, aspartate and glutamate metabolism (0.01 < p < 0.05).

In the present study, Metaboanalyst was utilized to identify the most relevant pathways and to elucidate the underlying mechanism of SYDS from the metabolic pathways from a global angle. The results revealed that the primary disturbed pathways in plasma and urine differential metabolites in response to SYDS was the citrate cycle (TCA cycle: succinic acid, L-malic acid, citric acid), glyoxylate and dicarboxylate metabolism (hydroxypyruvic acid, succinic acid, L-malic acid, citric acid), sphingolipid metabolism (sphinganine, sphingosine 1-phosphate, sphinganine 1-phosphate) (with an impact-value of p < 0.01); tryptophan metabolism (serotonin, 5-hydroxyindoleacetic acid, 4,6-dihydroxyquinoline, N-methylserotonin), alanine, aspartate and glutamate metabolism (N-acetyl-L-aspartic acid, succinic acid) (with an impact-value of 0.01 < p < 0.05) (Fig. 3).

### Correlation analysis between microbial and metabolic profiling

**Figure-4:**
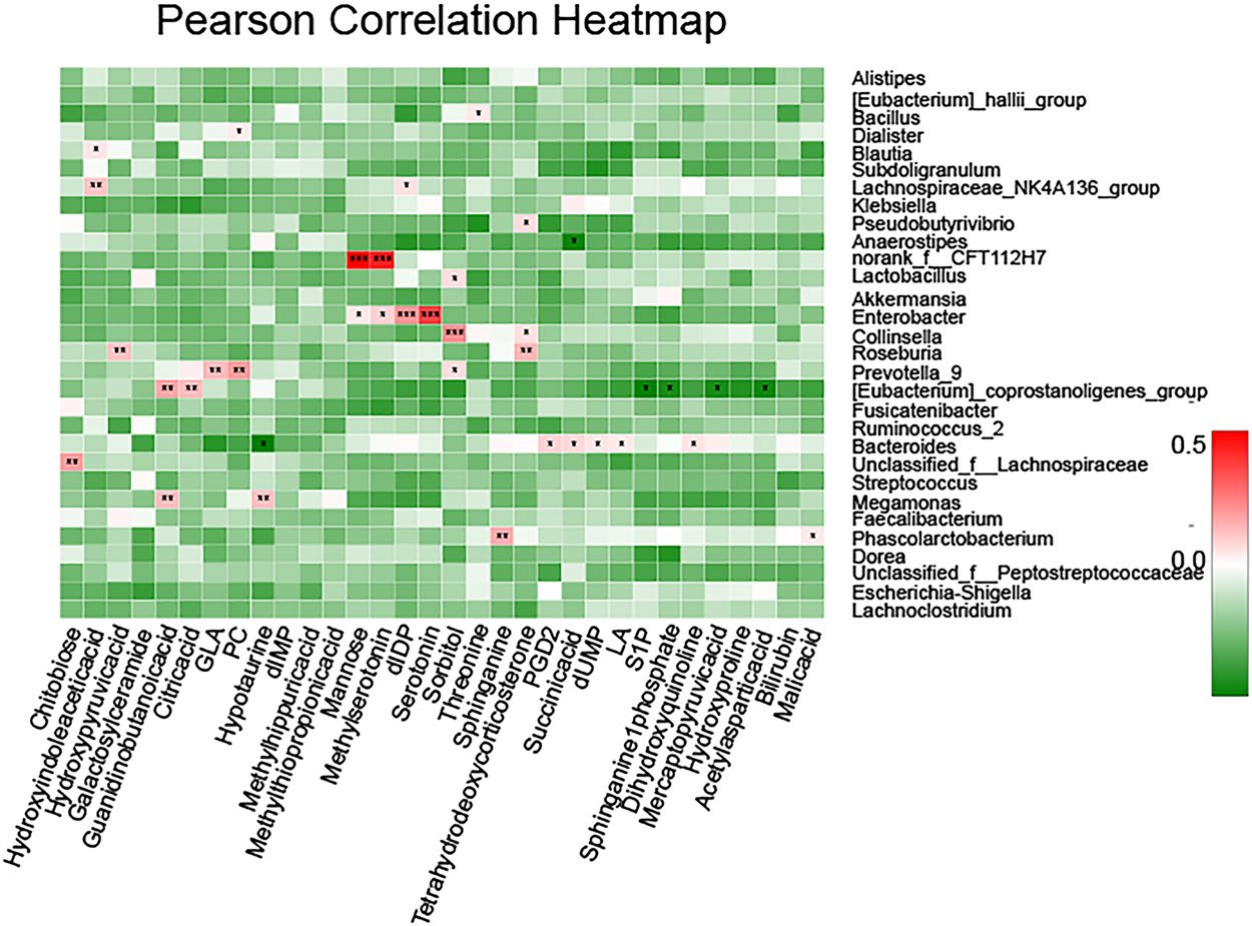
The correlation heatmap of identifying associations between the gut microbiota structure (genus level) and the differentiated metabolites (plasma, urine). The red color means positive correlation while the green color shows a negative correlation. The deeper color means the greater correlation (0.01 < p <  = 0.05 *, 0.001 < p <  = 0.01 **, p <  = 0.001 ***).

**Figure-5:**
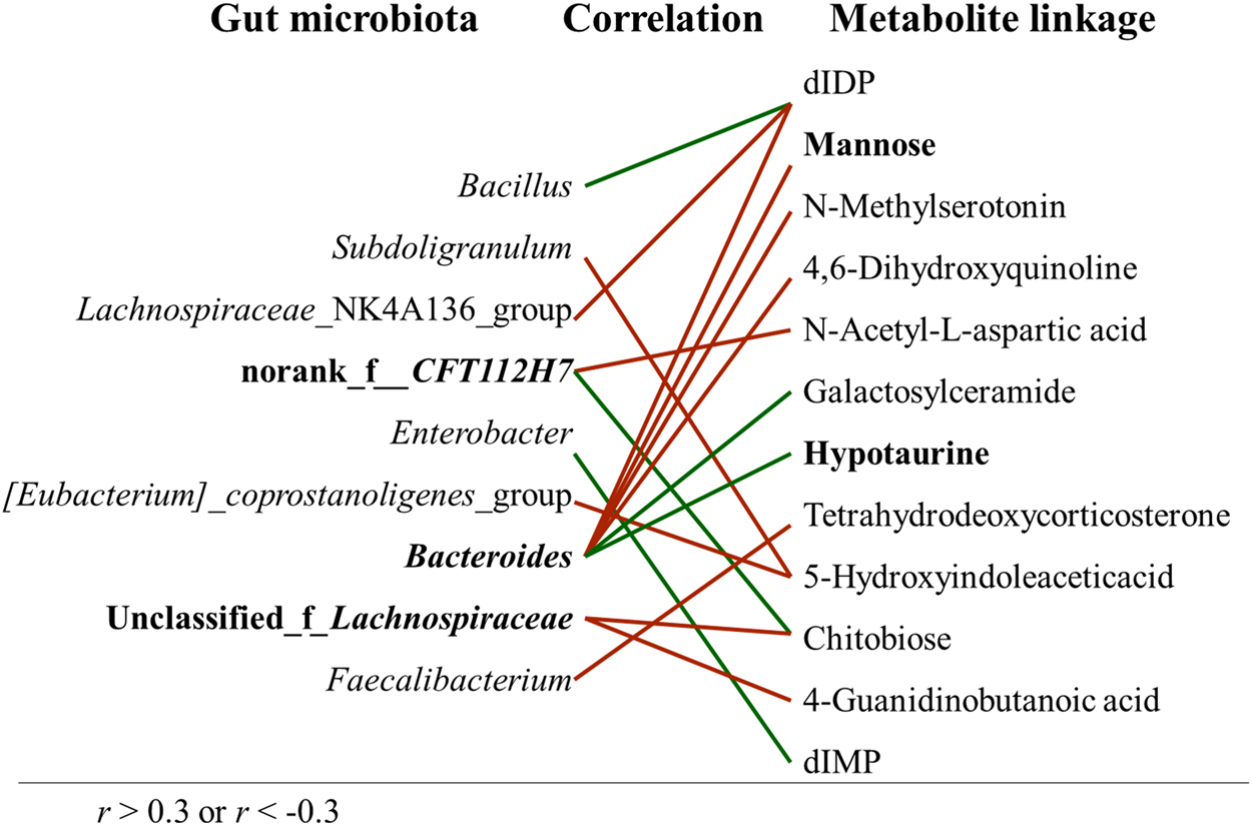
The gut microbiota, which was well predicted (|r| > 0.3) by the metabolic variation, is labeled with a similarity value. Associations with the metabolites are shown for each genus microbiota with the direction of correlation indicated by red (positive) or green (negative) lines.

As shown in Fig. 4, the covariation between UPLC-MS-based plasma and urine differentiated metabolites and 16 S rRNA data are presented in the form of a heatmap diagram. The metabolic associations of well predicted members in the gut microbiome (e.g., r > 0.3 or r < −0.3) are shown in Fig. 5. Some of the compounds are known as cometabolites and are produced by gut flora metabolism, such as hypotaurine and mannose. Some microbial members only revealed a sole metabolite association, for example, the connection between *bacillus* and dIDP (r = −0.41); other members had multiple connectivity, for example, norank_f_*CFT112H7*, was statistically linked with the presence of 2 metabolites including N-acetyl-L-aspartic acid (r = 0.38) and chitobiose (r = −0.34) while unclassified _f_*lachnospiraceae* was linked with the presence of 2 metabolites including chitobiose (r = 0.39) and 4-guanidinobutanoicacid (r = 0.34). Particularly, *bacteroides* was statistically linked with the presence of 6 metabolites namely mannose (r = 0.34), hypotaurine (r = −0.38), N-methylserotonin (r = 0.34), 4, 6-dihydroxyquinoline (r = 0.35), galactosylceramide (r = −0.35) and dIDP (r = 0.42).

## Discussion

TCM syndrome differentiation is the core of Chinese medicine theory because it is the first and most pivotal step for disease diagnosis and treatment. SYDS is a very common syndrome observed in TCM gastroenterology clinics. It is a complex phenotype ranging from effects on digestion to others affecting the circulatory system. In the opinion of Chinese medicine, TCM syndrome is a systematic and whole metabolic condition which is different from diseases in western medicine. gut microbiota are described as a hidden metabolic organ in the intestinal tract of the human. They are involved in digestion, absorption and metabolism in the human body. However, the structural characteristics of the gut microbiota and their interactions with the host metabolic phenotype in SYDS patients have scarcely been reported until now. In our study, an integrative LC-QTOF-MS based-metabolic and 16 S rRNA based microbial profiling was performed on a group of patients with SYDS and compared with matched healthy volunteers.

It was clear that the metabolic profile distributions of plasma/urine samples were distinctly different between the two groups. Through pathways enrichment analysis, it was demonstrated that the differentiated metabolites were mainly associated with disorders of energy and carbohydrate metabolism, lipid metabolism and gut microbiota-related tryptophan metabolism.

**Figure-6:**
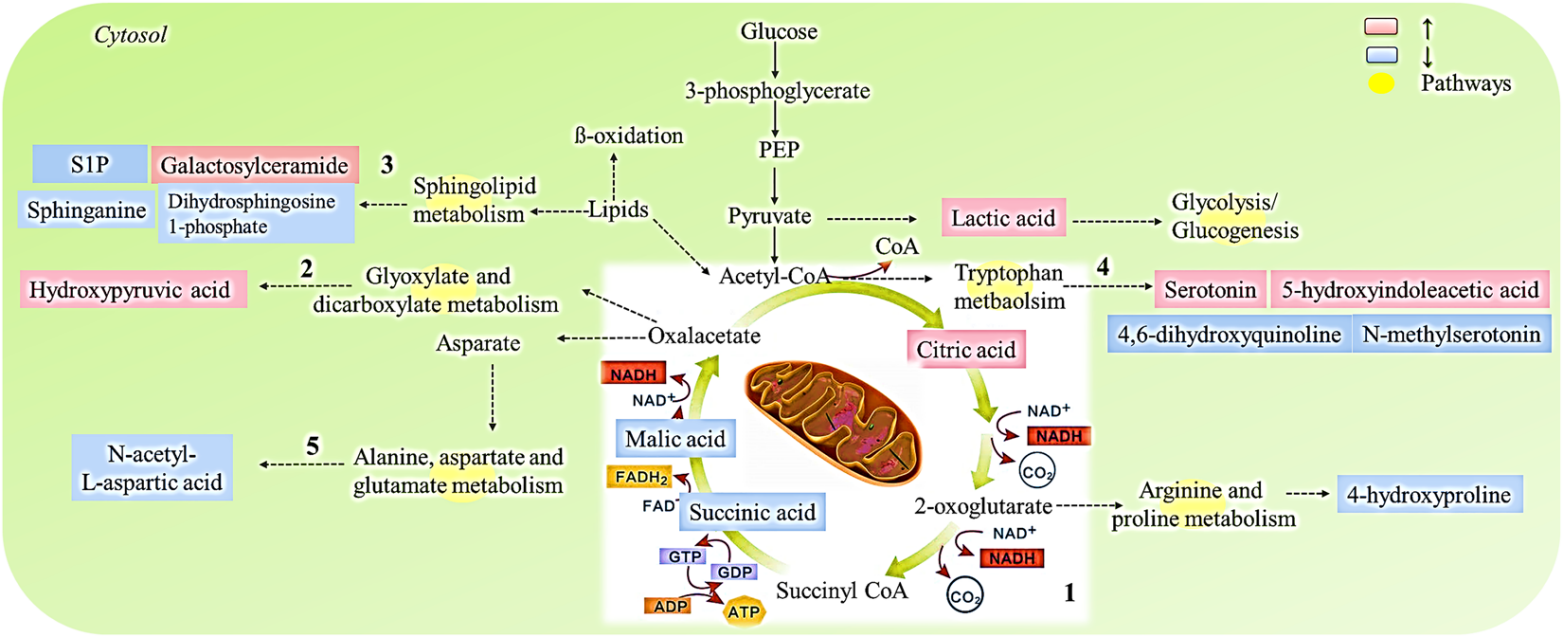
The energy metabolism and carbohydrate metabolism disorder in SYDS patients. (red means upregulated metabolites, blue means downregulated metabolites, yellow means metabolic pathways).

Energy metabolism and carbohydrate metabolism, the TCA cycle, and glyoxylate and dicarboxylate metabolism pathways pointed to a disorder of energy regulation (Fig. 6)^34,35^. The TCA cycle is an important energy production factory in all aerobic organisms through the oxidation of acetyl-CoA derived from three nutrients. Glyoxylate and dicarboxylate metabolism was associated with the TCA cycle via a connection with oxaloacetate. Citrate is an endogenous metabolite generated from glucose metabolism. In SYDS patients, the concentration of citric acid was significantly upregulated while succinic acid and L-malic acid levels were downregulated. The accumulation of citrate can reduce the activity of citrate synthase and thus affect the TCA cycle resulting in a reduced production of succinic acid and L-malic acid. Since the most efficient energy production factory of the TCA cycle was interrupted, aerobic glycolysis was activated which resulted in increased lactic acid levels, identified in SYDS plasma. This finding explains why SYDS patients generally have cold symptoms such as edema, cold limbs, a need to consume warm foodstuffs or feel uncomfortable after eating cold food^17^.

Moreover, metabolites in the TCA cycle can also provide many precursors of certain compounds that are used in numerous other biochemical reactions. For example, α-ketoglutarate is the precursor necessary for the synthesis of aspartic acid; aspartic acid can then be used to synthesize purine and pyrimidine bases. As a result, the related metabolites of N-acetyl-L-aspartic acid (alanine, aspartate and glutamate metabolism), dIMP, dIDP (purine metabolism) and dUMP (pyrimidine metabolism) also were significantly changed and detected as differentiated metabolites in SYDS patients but not in the healthy controls.

Lipid metabolism-related molecules, e.g. the sphingolipids, contain a lipophilic long-chain amino alcohol bound to a long-chain fatty acid by an amide linkage. Thus, the formation of the sphingosyl backbone depends on the amount of saturated fatty acids available. As an important energy source, lipid metabolism was also significantly perturbed in SYDS patients because of digestion system dysfunction.

The gut microbiota disorder-related metabolism of tryptophan was reported to be involved in gut microbiome-derived neurodevelopmental diseases^36^. The brain-gut axis is a two-way communication system between the central nervous system and the gut. Specifically, the intestinal microorganisms involved in tryptophan metabolism and the serotonin system may play a pivotal role. In our study, several metabolites of serotonin, including 5-hydroxyindoleacetic acid, 4, 6-dihydroxyquinoline and N-methylserotonin, were all derived from a tryptophan metabolism disorder in SYDS patients. Serotonin is a critical signalling molecule in the brain-gut microbiota axis^37^. Serotonin also has a stimulating effect on the gut by actions on the vagus nerve, ganglion cells and smooth muscle, which can increase gastrointestinal peristalsis and lead to the development of diarrhea. It is noteworthy that diarrhea was a typical clinical symptom observed in SYDS patients. N-methylserotonin is often found in urine specimens of patients with psychiatric disorders^38^. Therefore, the gut microbiota-mediated tryptophan metabolism indicated that SYDS patients might also suffer from neurodevelopmental disorders.

In studying microbiota profiles, the compositional structure and beta diversity analysis both revealed significant classifications between the groups. The Lefse difference analysis showed that the differentiated microbiota was mainly focused on the accumulated number of *firmicutes* and *clostridia* bacteria, which pointed to an energy metabolism dysfunction. By integrating the gut bacterial data with the identified differentiated metabolites, our results highlighted the tight crosstalk between the gut microbiota and host metabolism in SYDS patients. As shown in Fig. 5, the metabolic associations of each predicted genus bacteria were norank_f_*CFT112H7*, unclassified_f_*lachnospiraceae* and *bacteroides*. f_*lachnospiraceae* is the most significant n-butyrate producing microorganism in the gut with functions on host energy regulation and mucosal integrity^39^. When *bacteroides* were analysed, six metabolites were statistically correlated including mannose, hypotaurine, N-methylserotonin, 4, 6-dihydroxyquinoline, galactosylceramide and dIDP. In particular, two metabolites of mannose (r = 0.34) and hypotaurine (r = −0.38) attracted our attention because they were associated with the gut microbiota involved in host metabolism. *Bacteroides* spp. are known to decompose various indigestible dietary plant polysaccharides by producing enzymes similar to glycosyl transferases, glycoside hydrolases and polysaccharide lyases. It was reported that human gut *bacteroidetes* (*bacteroides thetaiotaomicron*) can make use of the food yeast mannan by generating large oligosaccharides that are subsequently depolymerized to mannose by the action of periplasmic enzymes^40^.

*Bacteroides* spp. can also participate in bile acid-related lipid metabolism^41–43^. Hypotaurine is enzymatically oxidized to yield taurine by hypotaurine dehydrogenase in the taurine and hypotaurine metabolism pathways in mammalians^44^. In the liver, taurine or glycine are conjugated with primary bile acids (cholic and chenodeoxycholic acids), stored in the gallbladder, and released into the duodenum after ingestion of fat^45^. In the intestine, bile acids are metabolized by bacteria to more hydrophobic bile acids species via 7-α-dehydroxylation and produce secondary bile acids (deoxycholic and lithocholic acid)^46^. Most of the bile acids are reabsorbed in the distal ileum and then recycled via the portal vein into the liver^47^. Therefore, changes in the gut microbiota strongly regulate bile acid metabolism, including modification of enterohepatic recycling of taurine conjugates to influence fat and cholesterol absorption in the intestinal tract (Fig. S3)^48^. The data correlation between *bacteroides* and hypotaurine found here also indirectly reflected these interactions. Moreover, the bile acid metabolism associated compound bilirubin was also found to be significantly downregulated (Table 2) in plasma taken from SYDS patients. Bilirubin is excreted in the bile and is responsible for the color of bile acid and the yellow discoloration seen in patients with jaundice. Its concentration in the human body reflects the balance between production and excretion. Research has indicated that in the absence of liver disease, individuals with high levels of total bilirubin may possess more health benefits than those with lower levels of bilirubin^49^. Therefore, these results suggest that bile acid-associated lipid metabolism in SYDS patients was also largely modified compared with the healthy control group.

In our study, data correlation further confirmed the connection between disturbances in the intestinal flora and the metabolic consequences of the perturbed microorganism-host metabolic balance in SYDS patients. Our constructed approach can also be applied to other gut microbiota-regulated TCM syndrome patterns or drug metabolism.

## Conclusions

In TCM, the types of syndrome are mainly diagnosed with symptom-based diagnostic criteria, which are usually subjective and experience-oriented. As a result, the elucidation on biological basis of TCM syndromes is urgently needed for promoting objectification of TCM diagnosis. SYDS is a very common syndrome in gastroenterology in TCM. Our current study provided preliminary evidence on the metabolic and microbial contribution to SYDS patients, which highlighted the significance and potential of combined metabolomics and 16 S rRNA sequencing approach in TCM syndrome study. The identification of the microbial species that have strong host metabolic connectivity can lay the foundations of gut microbiota community dysbiosis for SYDS. This research has also enriched the microecology of Chinese medicine and broadened our understanding of genome-related TCM syndrome development.

There are some limitations of present study. First, the identified differences in gut microbiota and metabolites for diagnostic and prognostic evaluations were not verified by further study. Secondly, the main data and results were obtained from small sample size and single-centre source, which may not be able to represent the real situation of the syndrome distribution and bring potential bias in results. Accordingly, the findings of current study warrant further investigation for uncovering the metabolic and microbial basis of SYDS in TCM.

## Electronic supplementary material

Supplementary material
