# Supplementary material for: Integrative metabolic and microbial profiling on patients with Spleen-yang-deficiency syndrome
Source: Sci Rep. 2018 Apr 26;8:6619. doi: 10.1038/s41598-018-24130-7 (PMC5920061; doi:10.1038/s41598-018-24130-7)
Supplement: Supplementary file 1 — Supplementary material [file 41598_2018_24130_MOESM1_ESM.pdf]

Integrative metabolic and microbial profiling on patients with spleen-yang-deficiency syndrome

Zhang Lin[a], Wu Ye[b], Xianpeng Zu[c], Haisheng Xie[c], Houkai Li[d], Yiping Li[b]*, Weidong Zhang[a,c, d]*

Supplementary material

Figure-S1. Shannon-rarefaction curve to evaluate the sequencing depth in each sample.

Figure-S2. OPLS-DA score plots showing the distributions of plasma/urine detected in both ESI+ and ESI- modes in SYDS patients (in red circle) and healthy individuals (in black star).

Figure-S3. The gut microbiota involved bile acid metabolism.

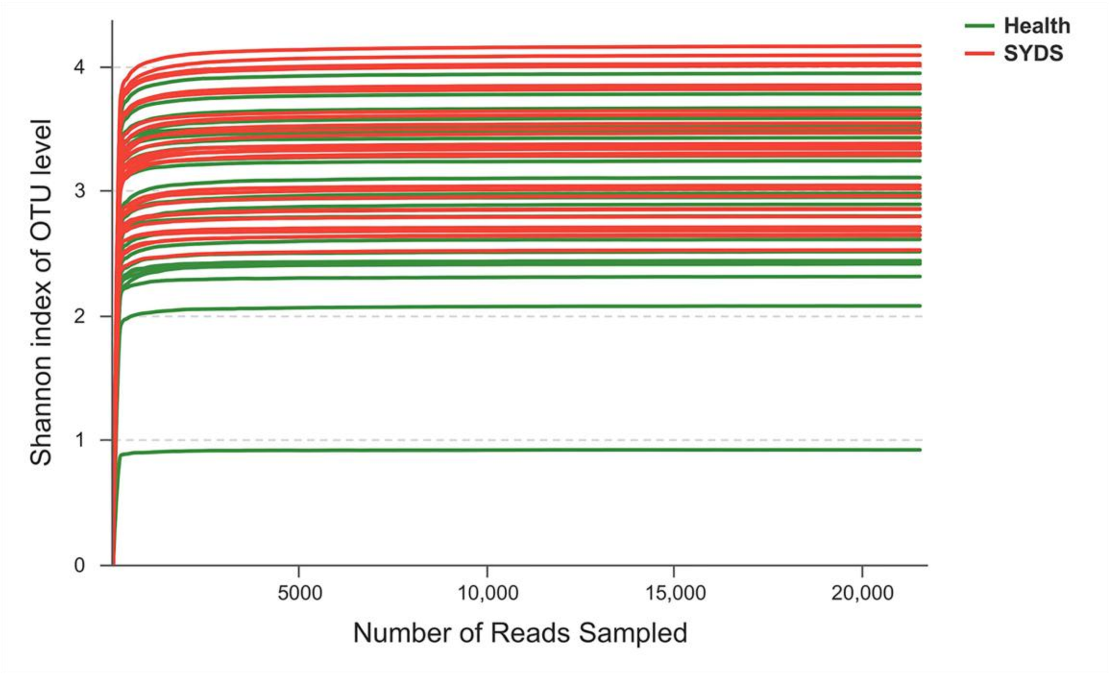
Health
SYDS
Shannon index of OTU level
0
1
2
3
4
5000
10,000
15,000
20,000
Number of Reads Sampled

Figure-S1

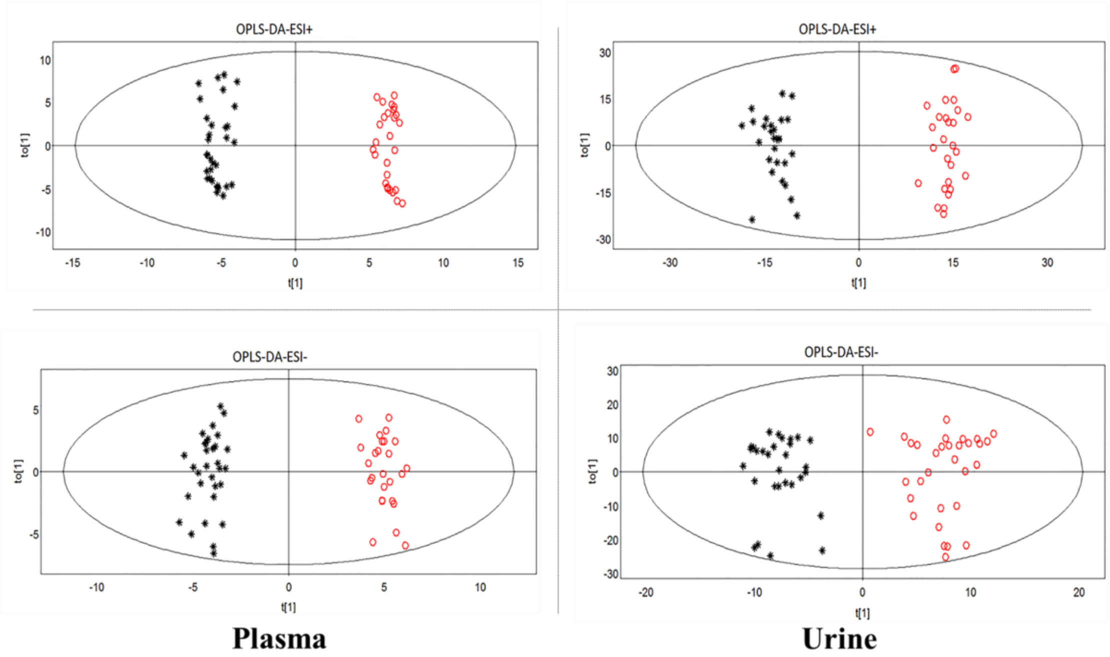

OPLS-DA-ESI+
OPLS-DA-ESI+
OPLS-DA-ESI-
OPLS-DA-ESI-
to[1]
t[1]
Plasma
Urine

Figure-S2

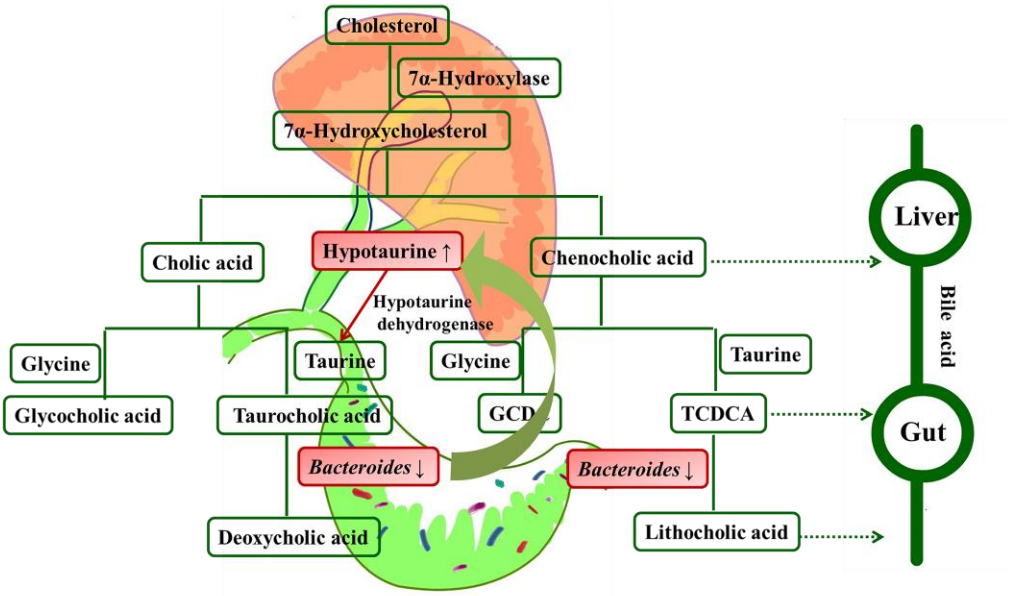

Cholesterol
7α-Hydroxylase
7α-Hydroxycholesterol
Cholic acid
Hypotaurine ↑
Hypotaurine
dehydrogenase
Chenocholic acid
Glycine
Taurine
Glycine
Taurine
Glycocholic acid
Taurocholic acid
GCDCA
TCDCA
Bacteroides ↓
Bacteroides ↓
Deoxycholic acid
Lithocholic acid
Liver
Bile acid
Gut

Figure-S3
